# Design, synthesis and biological potential of heterocyclic benzoxazole scaffolds as promising antimicrobial and anticancer agents

**DOI:** 10.1186/s13065-018-0464-8

**Published:** 2018-09-19

**Authors:** Saloni Kakkar, Sanjiv Kumar, Balasubramanian Narasimhan, Siong Meng Lim, Kalavathy Ramasamy, Vasudevan Mani, Syed Adnan Ali Shah

**Affiliations:** 1 Faculty of Pharmaceutical Sciences, Maharshi Dayanand University, Rohtak, 124001 India; 2 Faculty of Pharmacy, Universiti Teknologi MARA (UiTM), 42300 Bandar Puncak Alam, Selangor Darul Ehsan Malaysia; 3 Collaborative Drug Discovery Research (CDDR) Group, Pharmaceutical Life Sciences Community of Research, Universiti Teknologi MARA (UiTM), 40450 Shah Alam, Selangor Darul Ehsan Malaysia; 4 Department of Pharmacology and Toxicology, College of Pharmacy, Qassim University, Buraidah, 51452 Kingdom of Saudi Arabia; 5 Atta-ur-Rahman Institute for Natural Products Discovery (AuRIns), Universiti Teknologi MARA, 42300 Bandar Puncak Alam, Selangor Darul Ehsan Malaysia

**Keywords:** Benzoxazole molecules, Synthesis, Antimicrobial activity, Anticancer activity

## Abstract

**Background:**

Benzoxazole is the most important class of heterocyclic compound in medicinal chemistry. It has been incorporated in many medicinal compounds making it a versatile heterocyclic compound that possess a wide spectrum of biological activities.

**Results:**

The molecular structures of synthesized benzoxazole derivatives were confirmed by physicochemical and spectral means. The synthesized compounds were further evaluated for their in vitro biological potentials i.e. antimicrobial activity against selected microbial species using tube dilution method and antiproliferative activity against human colorectal carcinoma (HCT 116) cancer cell line by Sulforhodamine B assay.

**Conclusion:**

In vitro antimicrobial results demonstrated that compounds **4**, **5**, **7** and **16** showed promising antimicrobial potential. The in vitro anticancer activity indicated that compounds **4** and **16** showed promising anticancer activity against human colorectal cancer cell line (HCT 116) when compared to standard drug and these compounds may serve as lead compound for further development of novel antimicrobial and anticancer agents.
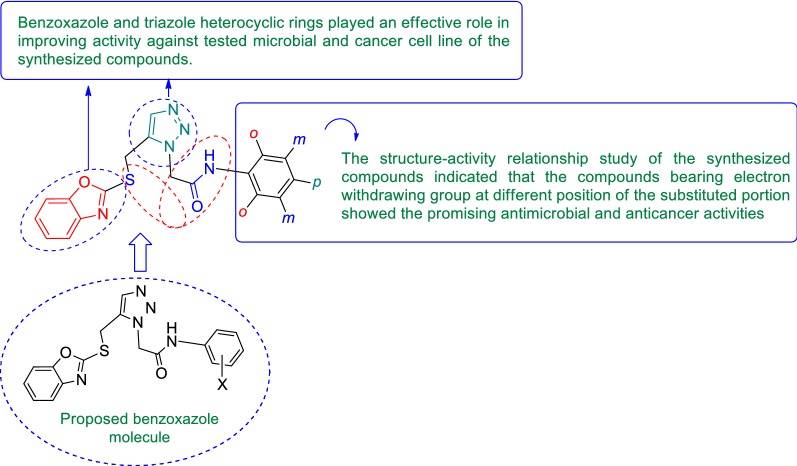

## Background

Colorectal cancer is one of the most dangerous forms of cancer, causing the deaths of many patients every year [1]. As such, a significant progress is being made continuously towards developing novel chemotherapeutic agents [2, 3]. One of the standard drugs for treatment of colorectal cancer is 5-fluorouracil (5-FU). However it is associated with a lot of side effects as it not only affects the cancer cells but also the normal cells [3–7]. In order to overcome the undesirable side effects of available anticancer agents there is a need to develop novel chemotherapeutic agents for more effective cancer treatment [2].

The number of cases of multidrug resistant bacterial infections is increasing at an alarming rate and clinicians have become reliant on vancomycin as the antibiotic for serious infections resistant to traditional agents which indicated that there is a need for the development of new classes of antimicrobial agents [8]. Hence there is a need to develop those agents whose chemical characteristics clearly differ from those existing agents and can overcome the problem of resistance [9].

**Fig. 1:**
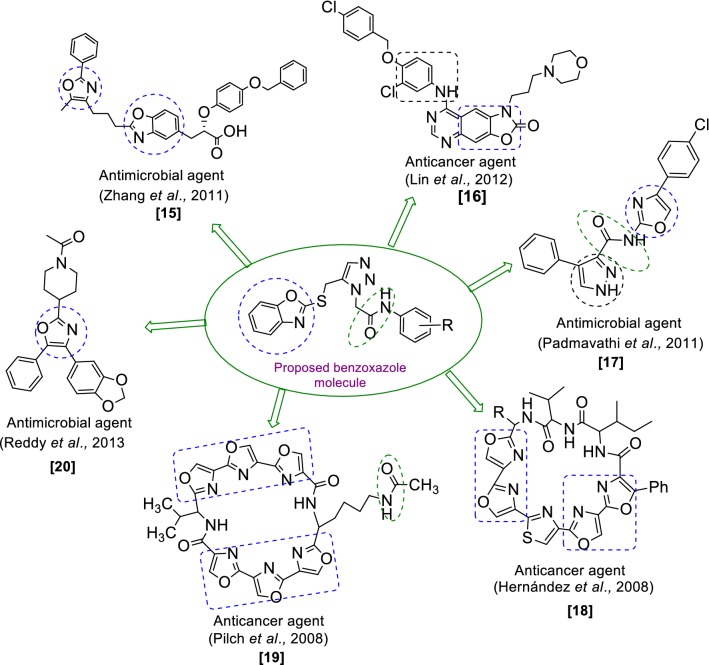
Design of benzoxazole molecules for antimicrobial and anticancer potential based on literature

Benzoxazole belongs to one of the most important class of heterocyclic compounds which are very significant for medicinal field. It has been incorporated in many medicinal compounds that made it versatile heterocyclic compound possessing wide spectrum of biological activities viz: antimicrobial [10, 11], analgesic/anti-inflammatory [12], antitumor [13], antidiabetic activity [14] etc. Keeping in view of the pharmacological importance of benzoxazole derivatives, the present study had synthesize some new benzoxazole derivatives and evaluate their antimicrobial and antiproliferative activities. The design of benzoxazole molecules with antimicrobial and anticancer potential was based on literature as shown in Fig. 1.

## Results and discussion

### Chemistry

**Scheme 1:**
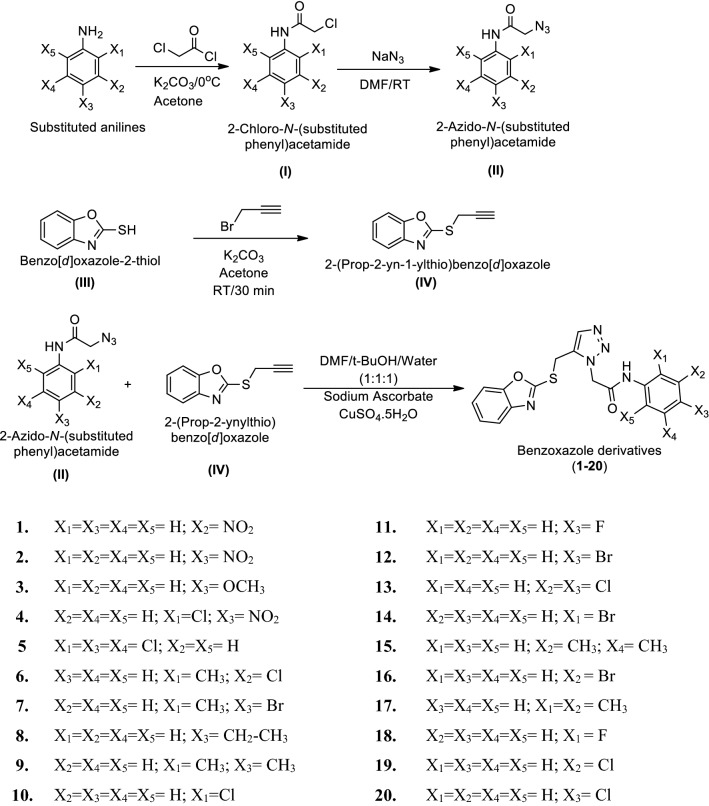
Synthesis of benzoxazole derivatives (**1**–**20**)

A series of benzoxazole derivatives (**1**–**20**) was synthesized using synthetic procedures as outlined in Scheme 1. Initially, 2-chloro-*N*-(substituted phenyl)acetamide (**I**) was prepared by reacting substituted aniline with chloroacetyl chloride in the presence of acetone and powdered potassium carbonate. To prepare 2-azido-*N*-(substituted phenyl)acetamide (**II**) reaction was carried out between **I** in dry DMF and sodium azide at room temperature. Benzo[*d*]oxazole-2-thiol (**III**) was prepared from 2-aminophenol in methanol, potassium hydroxide followed by the addition of carbon-di-sulphide. Further, to a solution of **III** in acetone was added anhydrous potassium carbonate powder followed by slow addition of 3-bromoprop-1-yne at 0 °C and the obtain 2-(prop-2-yn-1-ylthio)benzo[*d*]oxazole (**IV**). Finally, **II** and **IV** were dissolved in a mixture of t-BuOH:H_2_O:DMF followed by the addition of sodium ascorbate and copper (II) sulfate so as to obtain target benzoxazole derivatives (**1**–**20**). The synthesized compounds were confirmed by physicochemical properties (Table 1) i.e. melting point, molecular formula, R_f_ value, % yield and spectral interpretation details (Table 2) i.e. FT-IR, NMR and Mass, which are in agreement with the proposed molecular structures. The three obvious peaks in the IR spectra of the title compounds at 1689–1662 cm^−1^, 3315–2986 cm^−1^ and 1499–1408 cm^−1^ are attributed to C=N group of oxazole ring, C–H and C=C groups of aromatic ring, respectively. The absorption peak of C–F group in aromatic fluoro compounds (**11** and **18**) appeared at 1235–1207 cm^−1^ whereas bands at 738–622 cm^−1^ correspond to C–Br stretching of aromatic bromo derivatives (**7**, **12**, **14** and **16**). The presence of aryl alkyl ether group (C–O–C, Ar–OCH_3_) in compound **3** showed a band at 1194 cm^−1^. Further the presence of chloro group (Ar–Cl) in compounds **5**, **6**, **10**, **13**, **19** and **20** showed IR stretches at 744–739 cm^−1^. The IR band at 1653–1578 cm^−1^ indicated the presence of CONH group of synthesized compounds. The compounds **1**, **2** and **4** displayed IR stretching around 1394–1341 cm^−1^ that corresponds to C-N symmetric stretching of aromatic NO_2_ group.

**Table 1:** Physicochemical properties of synthesized benzoxazole derivatives

Comp.	Molecular mass	M. formula	m.p. °C	R_*f*_ value	% yield
**1:** 2-(5-((Benzo[*d*]oxazol-2-ylthio)methyl)-1*H*-1,2,3-triazol-1-yl)-*N*-(3-nitrophenyl)acetamide	410.41	C_18_H_14_N_6_O_4_S	152–154	0.17	76
**2:** 2-(5-((Benzo[*d*]oxazol-2-ylthio)methyl)-1*H*-1,2,3-triazol-1-yl)-*N*-(4-nitrophenyl)acetamide	410.41	C_18_H_14_N_6_O_4_S	165–167	0.18	81
**3:** 2-(5-((Benzo[*d*]oxazol-2-ylthio)methyl)-1*H*-1,2,3-triazol-1-yl)-*N*-(4-methoxyphenyl) acetamide	395.43	C_19_H_17_N_5_O_3_S	102–104	0.23	75
**4:** 2-(5-((Benzo[*d*]oxazol-2-ylthio)methyl)-1*H*-1,2,3-triazol-1-yl)-*N*-(2-chloro-4-nitrophenyl) acetamide	444.85	C_18_H_13_ClN_6_O_4_S	144–146	0.20	86
**5:** 2-(5-((Benzo[*d*]oxazol-2-ylthio)methyl)-1*H*-1,2,3-triazol-1-yl)-*N*-(2,4,5-trichlorophenyl) acetamide	468.74	C_18_H_12_Cl_3_N_5_O_2_S	189–191	0.21	79
**6:** 2-(5-((Benzo[*d*]oxazol-2-ylthio)methyl)-1*H*-1,2,3-triazol-1-yl)-*N*-(3-chloro-2-methylphenyl) acetamide	413.88	C_19_H_16_ClN_5_O_2_S	138–140	0.22	82
**7:** 2-(5-((Benzo[*d*]oxazol-2-ylthio)methyl)-1*H*-1,2,3-triazol-1-yl)-*N*-(4-bromo-2-methyl-phenyl) acetamide	458.33	C_19_H_16_BrN_5_O_2_S	127–129	0.22	85
**8:** 2-(5-((Benzo[*d*]oxazol-2-ylthio)methyl)-1*H*-1,2,3-triazol-1-yl)-*N*-(4-ethylphenyl)acetamide	393.46	C_20_H_19_N_5_O_2_S	118–120	0.23	85
**9:** 2-(5-((Benzo[*d*]oxazol-2-ylthio)methyl)-1*H*-1,2,3-triazol-1-yl)-*N*-(2,4-dimethylphenyl) acetamide	393.49	C_20_H_19_N_5_O_2_S	108–110	0.23	81
**10:** 2-(5-((Benzo[*d*]oxazol-2-ylthio)methyl)-1*H*-1,2,3-triazol-1-yl)-*N*-(2-chlorophenyl)acetamide	399.85	C_18_H_14_ClN_5_O_2_S	144–146	0.19	86
**11:** 2-(5-((Benzo[*d*]oxazol-2-ylthio)methyl)-1*H*-1,2,3-triazol-1-yl)-*N*-(4-fluorophenyl)acetamide	383.40	C_18_H_14_FN_5_O_2_S	119–121	0.19	90
**12:** 2-(5-((Benzo[*d*]oxazol-2-ylthio)methyl)-1*H*-1,2,3-triazol-1-yl)-*N*-(4-bromophenyl)acetamide	444.31	C_18_H_14_BrN_5_O_2_S	172–174	0.20	77
**13:** 2-(5-((Benzo[*d*]oxazol-2-ylthio)methyl)-1*H*-1,2,3-triazol-1-yl)-*N*-(3,4-dichlorophenyl) acetamide	434.30	C_18_H_13_Cl_2_N_5_O_2_S	169–171	0.19	80
**14:** 2-(5-((Benzo[*d*]oxazol-2-ylthio)methyl)-1*H*-1,2,3-triazol-1-yl)-*N*-(2-bromophenyl)acetamide	444.31	C_18_H_14_BrN_5_O_2_S	131–133	0.19	81
**15:** 2-(5-((Benzo[*d*]oxazol-2-ylthio)methyl)-1*H*-1,2,3-triazol-1-yl)-*N*-(3,5-dimethylphenyl) acetamide	393.46	C_20_H_19_N_5_O_2_S	125–127	0.23	79
**16:** 2-(5-((Benzo[*d*]oxazol-2-ylthio)methyl)-1*H*-1,2,3-triazol-1-yl)-*N*-(3-bromophenyl)acetamide	444.31	C_18_H_14_BrN_5_O_2_S	151–153	0.18	78
**17:** 2-(5-((Benzo[d]oxazol-2-ylthio)methyl)-1*H*-1,2,3-triazol-1-yl)-*N*-(2,3-dimethylphenyl) acetamide	393.46	C_20_H_19_N_5_O_2_S	133–135	0.24	82
**18:** 2-(5-((Benzo[*d*]oxazol-2-ylthio)methyl)-1*H*-1,2,3-triazol-1-yl)-*N*-(2-fluorophenyl)acetamide	383.40	C_18_H_14_FN_5_O_2_S	119–120	0.20	89
**19:** 2-(5-((Benzo[*d*]oxazol-2-ylthio)methyl)-1*H*-1,2,3-triazol-1-yl)-*N*-(3-chlorophenyl)acetamide	399.85	C_18_H_14_ClN_5_O_2_S	161–163	0.19	86
**20:** 2-(5-((Benzo[*d*]oxazol-2-ylthio)methyl)-1*H*-1,2,3-triazol-1-yl)-*N*-(4-chlorophenyl)acetamide	399.85	C_18_H_14_ClN_5_O_2_S	166–168	0.19	82

**Table 2:** Spectral data of synthesized compounds **(1–20)**

Comp.	FT-IR (KBr cm^−1^)									^1^H NMR (δ, DMSO)	^13^C NMR (δ, DMSO)	MS: *m/z*
	C–H str. (Ar)	C=C str. (Ar)	N=CH str.	C–N str.	C–O–C str.	C–H str.	CONH str.	C–S str.	Other str.			
**1**	3088	1477	1688	1262	1134	2834	1647	672	1350 NO_2_ str.	7.63–8.57 (m, 8H, Ar–H), 7.33 (s, 1H, –CH of triazole), 4.76 (s, 2H, –N–CH_2_), 7.95 (s, 1H, –NH)	164.9, 151.2, 147.9, 141.1, 139.4, 130.3, 125.7, 125.1, 124.4, 118.2, 113.3, 110.2, 52.3, 26.3	411
**2**	3133	1460	1685	1259	1194	2973	1649	687	1394 NO_2_ str.	7.66–8.25 [m, 8H, Ar–H), 7.34 (s, 1H, –CH of triazole), 4.75 (s, 2H, –N–CH_2_), 7.82 (s, 1H, –NH)]	165.2, 151.2, 144.4, 141.1, 124.6, 124.4, 118.3, 110.2, 52.3, 26.3	411
**3**	3138	1452	1662	1241	1178	2831	1618	674	–	6.88–7.59 (m, 8H, Ar–H), 4.26 (s, 2H, –CH_2_S), 7.32 (s, 1H, –CH of triazole), 4.77 (s, 2H, –N–CH_2_), 8.25 (s, 1H, –NH), 3.71 (s, 3H, –OCH_3_)	164.1, 155.4, 151.1, 140.8, 131.4, 124.7, 124.5, 120.7, 118.2, 113.9, 110.2, 55.1, 52.2, 26.3	396
**4**	3072	1451	1697	1275	1177	2887	1647	671	1341 NO_2_ str. 741 C–Cl str.	7.34–8.23 (m, 7H, Ar–H), 7.33 (s, 1H, –CH of triazole), 4.74 (s, 2H, –NCH_2_), 8.21 (s, 1H, –NH)	165.7, 151.3, 143.5, 141.1, 124.6, 124.3, 123.7, 118.3, 110.2, 52.3, 26.3	445
**5**	3077	1499	1689	1277	1181	–	1653	676	743 C–Cl str.	7.35–7.66 (m, 6H, Ar–H), 7.34 (s, 1H, –CH of triazole), 4.73 (s, 2H, –N–CH_2_), 8.09 (s, 1H, –NH)	165.3, 151.3, 141.1, 134.3, 130.6, 129.8, 124.9, 124.6, 124.3, 118.3, 110.2, 52.1, 26.3	469
**6**	3121	1499	1672	1272	1132	3067	1578	669	739 C–Cl str.	7.20–7.66 (m, 7H, Ar–H), 7.29 (s, 1H, –CH of triazole), 4.74 (s, 2H, –N–CH_2_), 8.22 (s, 1H, –NH), 2.51 (s, 3H, –CH_3_)	164.5, 151.2, 141.1, 136.9, 133.8, 130.3, 126.8, 124.6, 124.3, 118.3, 110.2, 51.9, 26.3	414
**7**	2986	1494	1674	1291	1185	2877	1601	742	622 C–Br str.	7.338–7.45 (m, 7H, Ar–H), 4.19 (s, 2H, –CH_2_S), 7.332 (s, 1H, –CH of triazole), 4.76 (s, 2H, –N–CH_2_), 8.24 (s, 1H, –NH), 2.51 (s, 3H, –CH_3_)	164.3, 151.1, 141, 134.1, 132.8, 128.8, 124.6, 118.2, 110.2, 52.1, 17.4	459
**8**	3080	1449	1662	1237	1130	2965	1606	709	–	7.14–7.66 (m, 8H, Ar–H), 7.31 (s, 1H, –CH of triazole), 4.74 (s, 2H, –N–CH_2_), 8.20 (s, 1H, –NH), 1.16 (s, 3H, –CH_3_), 2.58 (s, 2H, –CH_2_)	163.81, 151.3, 141.2, 136.1, 128.1, 124.6, 124.3, 118.3, 110.2, 52.2, 27.5, 15.5	394
**9**	3037	1486	1676	1293	1141	2993	1592	668	–	6.94–7.58 (m, 7H, Ar–H), 4.18 (s, 2H, –CH_2_S), 7.31 (s, 1H, –CH of triazole), 4.77 (s, 2H, –NCH_2_), 8.27 (s, 1H, –NH), 2.50 (s, 6H, (–CH_3_)_2_)	164.1, 151.1, 140.9, 134.6, 132.8, 131.4, 130.8, 126.5, 124.7, 124.4, 118.2, 110.2, 52.1, 26.3, 17.6	394
**10**	3121	1408	1675	1220	1137	2985	1641	675	743 C–Cl str.	7.15–7.58 (m, 8H, Ar–H), 7.34 (s, 1H, –CH of triazole), 4.74 (s, 2H, –NCH_2_), 8.21 (s, 1H, –NH);	164.1, 151.3, 141.2, 125.6, 124.6, 124.3, 118.3, 110.2, 52.1, 26.4	400
**11**	3048	1491	1677	1289	1132	2879	1589	686	1235 C–F str.	7.19–7.65 (m, 8H, Ar–H), 7.31 (s, 1H, –CH of triazole), 4.75 (s, 2H, –NCH_2_), 8.24 (s, 1H, –NH)	164.7, 151.3, 141.1, 134.1, 129.5, 124.6, 124.3, 118.3, 110.2, 52.1, 26.4	384
**12**	3018	1488	1682	1288	1178	2949	1597	753	738 C–Br str.	7.337–7.52 (m, 8H, Ar–H), 7.332 (s, 1H, –CH of triazole), 4.73 (s, 2H, –NCH_2_), 8.20 (s, 1H, –NH)	164.3, 151.3, 141.2, 137.7, 131.7, 124.6, 118.3, 110.2, 52.2, 26.4	445
**13**	3315	1470	1677	1291	1130	2992	1588	689	744 C–Cl str.	7.33–7.58 (m, 7H, Ar–H), 7.34 (s, 1H, –CH of triazole), 4.74 (s, 2H, –N–CH_2_), 8.21 (s, 1H, –NH)	164.7, 151.3, 141.2, 138.4, 131.1, 130.8, 124.6, 124.3, 120.4, 119.2, 110.2, 52.1, 26.4	435
**14**	3054	1495	1674	1286	1134	2880	1584	740	684 C–Br str.	7.14–7.60 (m, 8H, Ar–H), 7.31 (s, 1H, –CH of triazole), 4.73 (s, 2H, –NCH_2_), 8.21 (s, 1H, –NH)	164.6, 151.3, 141.1, 132.7, 128.1, 126.8, 124.6, 124.3, 118.3, 110.2, 51.9, 26.3	445
**15**	3144	1496	1676	1261	1179	3001	1607	687	–	6.7–7.33 (m, 7H, Ar–H), 7.34 (s, 1H, –CH of triazole), 4.73 (s, 2H, –NCH_2_), 8.18 (s, 1H, –NH), 2.50 (s, 6H, (–CH_3_)_2_)	163.9, 151.3, 141.1, 138.1, 137.8, 125.5, 124.6, 124.3, 118.3, 110.2, 52.2, 26.3, 21.1	394
**16**	3308	1475	1681	1226	1132	–	1619	743	676 C–Br str.	7.29–7.91 (m, 8H, Ar–H), 7.30 (s, 1H, –CH of triazole), 4.75 (s, 2H, –N–CH_2_), 8.24 (s, 1H, –NH)	164.5, 151.3, 141.1, 139.8, 130.9, 126.3, 124.6, 124.3, 121.5, 118.3, 110.2, 52.2, 26.4	445
**17**	3016	1460	1671	1211	1133	2947	1585	737	–	7.02–7.34 (m, 7H, Ar–H), 7.14 (s, 1H, –CH of triazole), 4.72 (s, 2H, –N–CH_2_), 8.18 (s, 1H, –NH), 2.50 (s, 6H, (–CH_3_)_2_)	164.3, 151.3, 141.2, 137.1, 130.9, 127.2, 125.5, 124.6, 124.3, 123.2, 118.3, 110.2, 51.9, 26.4, 13.9	394
**18**	3124	1454	1679	1293	1149	3057	1616	711	1207 C–F str.	7.16–7.67 (m, 8H, Ar–H), 7.29 (s, 1H, –CH of triazole), 4.73 (s, 2H, –N–CH_2_), 8.20 (s, 1H, –NH)	164.7, 163.6, 151.3, 141.2, 125.6, 124.6, 124.4, 124.3, 123.6, 118.3, 115.4, 110.2, 51.9, 26.4	384
**19**	3310	1467	1678	1271	1130	3136	1595	680	742 C–Cl str.	7.14–7.66 (m, 8H, Ar–H), 7.33 (s, 1H, –CH of triazole), 4.73 (s, 2H, –NCH_2_), 8.20 (s, 1H, –NH)	164.5, 151.3, 142.2, 141.2, 133.1, 130.6, 124.6, 124.3, 123.4, 118.3, 110.2, 52.1, 26.4	400
**20**	3126	1488	1670	1275	1148	3041	1589	671	739 C–Cl str.	7.31–7.59 (m, 8H, Ar–H), 7.33 (s, 1H, –CH of triazole), 4.73 (s, 2H, –NCH_2_), 8.22 (s, 1H, –NH)	164.2, 151.3, 141.2, 137.3, 128.8, 124.6, 124.3, 118.3, 110.2, 52.2, 26.4	400

Str.: stretching, Ar: aromatic

In ^1^H-NMR spectra the multiplet signals between 6.70 and 8.57 ppm are assigned to the presence of aromatic protons of synthesized compounds (**1**–**20**). The compound **3** showed a singlet at 3.71 ppm due to the existence of –OCH_3_ of Ar–OCH_3_ in its structure. All the synthesized compounds showed a singlet at 7.34–7.14 ppm which corresponds to the presence of N–CH of triazole. Compounds, **6**, **7**, **9**, **15** and **17** showed singlet around 2.50 ppm due to the existence of –CH_3_ group at *ortho* and *para* position. The appearance of singlet at 4.72–4.77 ppm and 8.27–7.82 ppm are due to –CH_2_ and –NH group, respectively. ^13^C-NMR spectral data showed the confirmation of carbon atom in the assigned molecular structures of the synthesized compounds. The mass spectra of title compounds shows consistency between [M]^+^ ion absorption signal and the calculated molecular weight. The synthesized benzoxazole derivatives (**1**–**20**) were screened for their pharmacological activity i.e. antimicrobial and antiproliferative activities against selected microbial (bacterial and fungal) organisms and cancer cell line (HCT 116), respectively (using standard protocol shown in experimental section). The structure–activity relationship study of the synthesized compounds indicated that the compounds bearing electron withdrawing group at different position of the substituted portion showed the promising antimicrobial and anticancer potentials.

#### In vitro antimicrobial activity

**Fig. 2:**
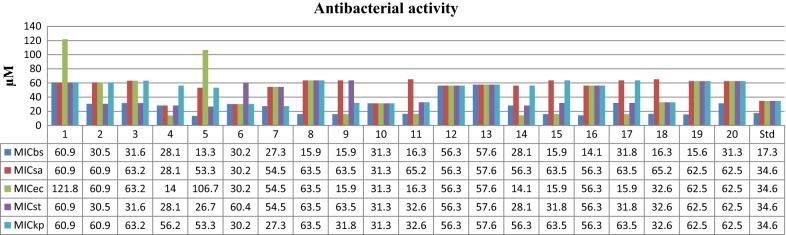
Antibacterial screening results of the synthesized benzoxazole derivatives

**Fig. 3:**
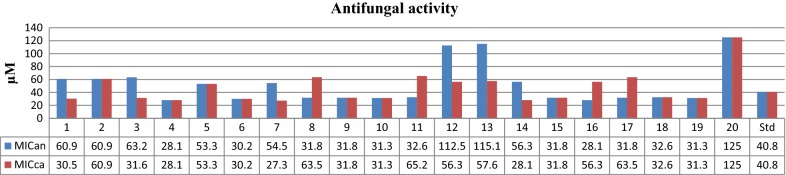
Antifungal screening results of the synthesized benzoxazole derivatives

The synthesized benzoxazole compounds (**1**–**20**) were investigated for their antimicrobial potential against selected Gram-positive (*S. aureus, B. subtilis*), Gram-negative (*E. coli*, *K. pneumoniae*, *S. typhi)* bacterial and fungal (*C. albicans, A. niger*) organisms by tube dilution method (Table 3, Figs. 2 and 3). In case of Gram-positive bacteria, compound **5** (MIC_*bs*_= 13.3 µM and MIC_*st*_= 26.7 µM) showed the significant activity against *B. Subtilis* and *S. typhi,* respectively. Other side, compound **4** (MIC_*sa, an*_= 28.1 µM and MIC_*ec*_ = 14 µM) showed promising activity against *S. aureus*, *A. niger* and *E. coli,* respectively. Compound **7** (MIC_*kp,* ca_ = 27.3 µM) exhibited good activity against *K. pneumoniae* and *C. albicans.* Whereas, compound **16** was found to be most active one against *A. niger* with MIC value of 28.1 µM. In this series compound **4** having high antimicrobial potential among the synthesized compounds may be taken as lead compound for the development of novel antimicrobial agent.

**Table 3:** In vitro antimicrobial activity of the synthesized compounds

Compound no.	Antimicrobial results (MIC = µM)						
	Bacterial species					Fungal species	
	*BS*	*SA*	*EC*	*ST*	*KP*	*AN*	*CA*
**1**	60.9	60.9	121.8	60.9	60.9	60.9	30.5
**2**	30.5	60.9	60.9	30.5	60.9	60.9	60.9
**3**	31.6	63.2	63.2	31.6	63.2	63.2	31.6
**4**	28.1	28.1	14.0	28.1	56.2	28.1	28.1
**5**	13.3	53.3	106.7	26.7	53.3	53.3	53.3
**6**	30.2	30.2	30.2	60.4	30.2	30.2	30.2
**7**	27.3	54.5	54.5	54.5	27.3	54.5	27.3
**8**	15.9	63.5	63.5	63.5	63.5	31.8	63.5
**9**	15.9	63.5	15.9	63.5	31.8	31.8	31.8
**10**	31.3	31.3	31.3	31.3	31.3	31.3	31.3
**11**	16.3	65.2	16.3	32.6	32.6	32.6	65.2
**12**	56.3	56.3	56.3	56.3	56.3	112.5	56.3
**13**	57.6	57.6	57.6	57.6	57.6	115.1	57.6
**14**	28.1	56.3	14.1	28.1	56.3	56.3	28.1
**15**	15.9	63.5	15.9	31.8	63.5	31.8	31.8
**16**	14.1	56.3	56.3	56.3	56.3	28.1	56.3
**17**	31.8	63.5	15.9	31.8	63.5	31.8	63.5
**18**	16.3	65.2	32.6	32.6	32.6	32.6	32.6
**19**	15.6	62.5	62.5	62.5	62.5	31.3	31.3
**20**	31.3	62.5	62.5	62.5	62.5	125.0	125.0
**Ofloxacin**	17.3	34.6	34.6	34.6	34.6	–	–
**Fluconazole**	–	–	–	–	–	40.8	40.8

BS: *Bacillus subtilis*; SA: *Staphylococcus aureus*; EC: *Escherichia coli*; ST: *Salmonella typhi*; KP: *Klebsiella pneumoniae*; AN: *Aspergillus niger*; CA: *Candida albicans*

#### In vitro anticancer activity

The antiproliferative activity of the benzoxazole derivatives was assessed against the human colorectal cancer cell line (HCT 116 (ATCC CCL-247). Antiproliferative  screening results (Table 4) revealed that compounds **4** (IC_50_ = 22.5 µM) and **16** (IC_50_ = 38.3 µM) displayed most promising antiproliferative activity in reference to the standard drug 5-fluorouracil (IC_50_ = 12.2 µM).

**Table 4:** Anticancer activity results of synthesized compounds

Anticancer screening results (IC_50_ = µM)			
Compound no.	Cancer cell line (HCT 116)	Compound no.	Cancer cell line (HCT 116)
**1**	97.5	**11**	130.4
**2**	73.1	**12**	> 225.1
**3**	108.7	**13**	> 230.3
**4**	22.5	**14**	90.0
**5**	85.3	**15**	40.7
**6**	84.6	**16**	38.3
**7**	72.0	**17**	177.9
**8**	> 254.2	**18**	148.7
**9**	66.1	**19**	50.0
**10**	175.1	**20**	200.1
**5-Fluorouracil**	12.2	**5-Fluorouracil**	12.2

### Structure activity relationship (SAR)

**Fig. 4:**
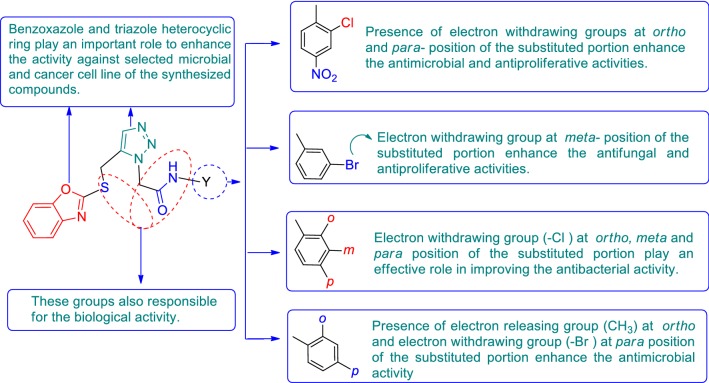
Structure activity relationship of benzoxazole derivatives

The structure activity relationship for antimicrobial and anticancer activities of synthesized benzoxazole derivatives (SAR, Fig. 4) can be deduced as follows:

- Presence of two heterocyclic moieties i.e. benzoxazole and triazole in the synthesized compounds, showed the promising in vitro antimicrobial and anticancer activities against the selected microbial organisms and cancer cell line, respectively.
- Presence of electron withdrawing groups (Cl and NO_2_) at *ortho* and *para*-positions, respectively of the substituted portion (Compound **4**), enhanced the antimicrobial activity against *S. aureus, E. coli*, *A. niger* and antiproliferative activity against  HCT 116 cancer cell line.
- Presence of electron releasing group (CH_3_) at *ortho* and electron withdrawing group (Br) at *para*-position of the substituted portion (Compound **7**) enhanced the antimicrobial activity against *K. pneumoniae* and *C. albicans.*
- Electron withdrawing group (Br) at *meta*-position of the substituted portion (Compound **16**), enhanced the antifungal and antiproliferative activities against *A. niger* and HCT 116 cancer cell line, respectively, as well as compound **5** have electron withdrawing group (Cl) at *ortho* and *para*-position of the substituted portion played an effective role in improving the antibacterial activity against *B. subtilis* and *S. typhi*.

The structure–activity relationship of the synthesized benzoxazole derivatives indicated that the compounds bearing electron withdrawing and electron releasing groups at different position of the substituted portion plays an excellent role in improving the antimicrobial and antiproliferative activities. The aforementioned facts are supported by the earlier research findings [21–23].

## Experimental section

### Material and reagents

The materials required to carry out this research work were obtained from commercial sources and were used with no further purification. Reaction monitoring was carried by thin-layer chromatography using 0.25 mm silica gel plates, using chloroform and methanol (9:1) as mobile phase and iodine vapours helped in observing the spots which were visualized in UV light. Melting point of compounds was determined by open capillary tube technique. An infrared spectrum was recorded (ATR, cm^−1^) in Bruker 12060280, software: OPUS 7.2.139.1294 spectrometer. ^1^H-NMR and ^13^C-NMR were recorded at 600 and 150 MHz, respectively on Bruker Avance III 600 NMR spectrometer by appropriate deuterated solvents. The results are conveyed in parts per million (*δ*, ppm) downfield from tetramethylsilane (internal standard). ^1^H-NMR spectral details of the synthesized derivatives are represented with multiplicity like singlet (s); doublet (d); triplet (t); multiplet (m) and the number hydrogen ion. Waters Micromass Q-ToF Micro instrument was utilized for obtaining the Mass spectra.

### General procedure for synthesis of benzoxazole derivatives (1–20)

#### Step A: Synthesis of 2-chloro-*N*-(substituted phenyl)acetamide derivatives (I)

To a stirred solution of substituted aniline (10 mmol) in acetone (35 ml) at 0 °C was added powdered potassium carbonate (50 mmol). After stirring the mixture for 30 min at 0 °C, chloroacetyl chloride (20 mmol) was added dropwise with vigorous stirring. The mixture was then continuously stirred at room temperature for 3 h. The mixture was then poured into water (400 ml) with stirring. The separated solid was filtered and washed with hexane (50 ml) to give the desired intermediate **I** in good yield.

#### Step B: Synthesis of 2-azido-*N*-(substituted phenyl)acetamide derivatives (II)

To a stirred solution of **I** (3.0 mmol) in dry DMF (15 ml) was slowly added sodium azide (6.0 mmol). The resulting reaction mixture was then stirred for 12 h at room temperature. The mixture was then poured into ice cold water (100 ml) with stirring. The separated solid was filtered and washed with water (50 ml) to give the desired compound **II** in good yield.

#### Step C: Synthesis of benzo[d]oxazole-2-thiol (III)

To a solution of 2-aminophenol (100 mmol) in methanol (150 ml) was added aqueous potassium hydroxide (130 mmol) in water (30 ml), followed by addition of carbon-di-sulfide (150 mmol). Resulting mixture was refluxed at 65 °C for 5 h. After the completion of reaction, reaction mixture was poured in water (500 ml), which was neutralized with conc. hydrochloric acid and the solid separated was filtered and washed with hexane to afford the pure compound **III** (Yield: 90%). MP: 168–170 °C.

#### Step D: Synthesis of 2-(prop-2-ynylthio)benzo[d]oxazole (IV)

To a solution of **III** (50 mmol) in acetone (150 ml) was added anhydrous potassium carbonate powder (100 mmol) with stirring. After 5 min, propargyl bromide (55 mmol) was added slowly at 0 °C and allowed to stir for 30 min at room temperature. After completion of the reaction, followed by TLC, the mixture was quenched with ice cold water (500 ml) with vigorous stirring. The solid product separated was filtered followed by washing with water (50 ml) which afforded the desired intermediate **IV** (Yield: 8.7 g, 92%). MP: 188–190 °C.

#### Step E: Synthesis of target compounds (1–20)

The intermediates **IV** (1.5 mmol) and **II** (1.5 mmol) were dissolved in a mixture of t-BuOH:H_2_O:DMF mixture (6 ml, 1:1:1). Sodium ascorbate (0.75 mmol) was added, followed by copper (II) sulfate (0.3 mmol). The mixture was stirred vigorously at room temperature until TLC indicated the disappearance of the starting materials (30 min). After completion of the reaction as monitored by TLC (CHCl_3_:MeOH/9:1, Rf: 0.17), solid separated in the reaction mass was then filtered and washed with water (10 ml) followed by methanol (10 ml) to give pure benzoxazole derivatives.

#### In vitro antimicrobial assay

The antimicrobial testing of the benzoxazole derivatives (**1**–**20**) was done by tube dilution method [24] against ofloxacin (antibacterial) and fluconazole (antifungal) as standard drugs using Gram-positive (*B. Subtilis* MTCC-441; *S. aureus,* MTCC-3160) and Gram-negative bacteria (*E. coli,* MTCC-443; *S. typhi,* MTCC-98; *K. pneumoniae,* MTCC-530). The antifungal activity was assayed against (*C. albicans,* MTCC-227) and mould (*A. niger*, MTCC-281). Serial dilutions of the test compounds and reference drugs were prepared in double strength nutrient broth I.P. (bacteria) or sabouraud dextrose broth I.P. (fungi) [25]. The stock solution of the test and reference compounds was prepared in dimethyl sulfoxide. The samples were incubated at 37 ± 1 °C for 24 h (bacteria), at 25 ± 1 °C for 7 days (*A. niger*) and at 37 ± 1 °C for 48 h (*C. albicans*), respectively and the results were recorded in terms of MIC. The MIC was the lowest concentration of the tested compound that yields no visible growth of microorganisms in the test tube.

### In vitro anticancer assay

The antiproliferative effect of benzoxazole derivatives was determined against the human colorectal carcinoma [HCT 116] cancer cell line  using the Sulforhodamine-B (SRB) assay. HCT 116 was seeded at 2500 cells/well (96 well plate). The cells were allowed to attach overnight before being exposed to the respective compounds (0.001–100 µg/mL) for 72 h. The highest concentration of each compound tested (100 µg/ml) contained only 0.1% DMSO (non-cytotoxic). SRB assay [26] was then performed. Trichloroacetic acid was used to fix the cell. Staining with 0.4% (w/v) Sulforhodamine B mixed with 1% acetic acid was performed for 30 min. After five washes of 1% acetic acid solution, protein-bound dye was extracted with 10 mM tris base solution. Optical density was read at 570 nm and IC_50_ (i.e. concentration required to inhibit 50% of the cells) of each compound was determined. Data was presented as mean IC_50_ of at least triplicates.

## Conclusion

In this study, new benzoxazole derivatives were designed and synthesized. These benzoxazole derivatives were evaluated for their biological potentials (antimicrobial and anticancer). In vitro antimicrobial results demonstrated that compounds **5**, **4**, **7** and **16** showed most promising antimicrobial activity against selected microbial species in reference to the standard drugs and in vitro antiproliferative  screening results indicated that compounds **4** and **16** showed promising anticancer potential against human colorectal cancer cell line in reference to the standard drugs. These compounds may serve as lead compounds for further development into novel antimicrobial and anticancer agents.
